# Neutrophil-to-Lymphocyte Ratio in Ovarian Cancer Patients with Low CA125 Concentration

**DOI:** 10.1155/2019/8107906

**Published:** 2019-06-25

**Authors:** Hongyan Zhang, Qianyu Huo, Lunhui Huang, You Cheng, Yunde Liu, Huijing Bao

**Affiliations:** ^1^School of Laboratory Science, Tianjin Medical University, Tianjin 300203, China; ^2^The Department of Laboratory Science, Tianjin Anding Hospital, Tianjin 300222, China; ^3^The Integrative Medical Diagnosis Laboratory, Tianjin Nankai Hospital, 300100, China

## Abstract

Ovarian cancer cases with low CA125 concentration are problematic and increase the high false negative results ratio during routine physical examination testing. Unfortunately, patients without early discovery have very low survival rates. In our study, we investigated the possible role of differential leukocyte counts and the neutrophil-to-lymphocyte ratio (NLR) in ovarian cancer patients to identify an additional discriminative marker to avoid missing diagnoses in normal physical examinations. One hundred seventy-three patients with epithelial ovarian cancer and 70 healthy controls were involved in our study. Based on the results, compared with the healthy controls, NLR was significantly different both in the low CA125 concentration group and in the complete patient group, indicating that NLR could be an effective marker for ovarian cancer screening. According to ROC, sensitivity, specificity, and NPV results, CA125 >35 U/ml is a good indicator for cancer in routine physical examination. However, in patients with low CA125 concentration, the CA125>7.65 U/ml and NLR >1.72 group yielded increased sensitivity with appropriate specificity and higher NPV results than the CA125 >35 U/ml group. We believe CA125>7.65 U/ml and NLR >1.72 should be effective makers for patients with low CA125 concentration. As a more sensitive and cost-effective strategy, this method could be conducted during routine ovarian cancer screening.

## 1. Introduction

Ovarian cancer is the most lethal gynecological cancer for women due to masking of symptoms in early stage disease. The majority of patients are diagnosed in late stage with a very low 5-year survival rate. However, if patients can be diagnosed in early stage, this number could be increased by 90% [1], so early diagnosis is very important for patient survival. CA125, also known as MUC16, was proposed as a serum biomarker for ovarian cancer in 1983. It is a high molecular weight glycoprotein expressed by 80% of epithelial ovarian cancers [2]. The concentration of serum CA125 is also correlated with disease stage and recurrence, as well as patient survival.

Currently, a combination of serum CA125 level and imaging tests is used for ovarian cancer diagnosis, and serum CA125 alone is used for ovarian cancer screening in routine physical examination tests. Thirty-five kilounits/L is the cutoff value of serum CA125 concentration for ovarian cancer screening and assisting clinical diagnosis. However, not all ovarian cancer patients exhibit perfect diagnostic results during physical examination for ovarian cancer screening, and approximately 20% of women with the cancer have concentrations of serum CA125 lower than 35 kilounits/L [3]. Due to these problematic low CA125 concentration cases, high false negative results are obtained during cancer screening, which does not facilitate early diagnosis of ovarian cancer.

Tumor cells can be both suppressed and stimulated by inflammatory cells, and preoperative inflammatory markers, such as neutrophil-to-lymphocyte ratio (NLR), have been studied in many cancers [4–6]. In ovarian cancer, NLR is associated with epithelial ovarian cancer and can be used to predict survival after treatment [7, 8]. A high NLR is correlated with poor prognosis [9]. All of these results indicate that NLR may be another good indicator for use in ovarian cancer screening.

Based on these findings, identifying a way to avoid false negative results due to low CA125 concentration seems urgent, especially in ovarian cancer screening during routine physical examination testing. In this study, we investigated the possible role of differential leukocyte counts (neutrophils, lymphocytes, and monocytes) and NLR in ovarian cancer screening. Based on all the data discussed in this paper, we want to find an additional discriminative marker for ovarian cancer screening, especially to avoid missing diagnoses in normal physical examination testing.

## 2. Materials and Methods

### 2.1. Study Subjects

One hundred seventy-three cases of epithelial ovarian cancer were collected between January 2012 and February 2014 in Tianjin Tumor Hospital, and diagnosis was confirmed by pathological assessment. Clinical and pathological information for these patients was recorded at initial diagnosis. Preoperative blood count values for patients, such as absolute neutrophil value, absolute lymphocyte value, absolute monocyte value, and NLR, were also recorded and calculated for this study. NLR is defined as the absolute neutrophil count divided by the absolute lymphocyte count. The International Federation of Gynecology and Obstetrics (FIGO) stage, histological type, and pathological grade, based on the criteria of the FIGO and the World Health Organization, were established in all cases. Seventy healthy controls were selected for physical examination in the same hospital, ranging from 28 to 71 years old. All samples with infectious diseases, blood system diseases, thrombosis, and hemorrhage, which affect blood routine results, were excluded.

### 2.2. Statistical Analysis

All statistical analyses were performed using the statistical software SPSS 18.0 and MedCalc 15.8. Data are summarized as the number of observations, the median, and the range. The Mann-Whitney U test was used to assess differences in continuous variables among the three groups. To quantify the correlation between categorical variables and continuous clinical variables, Spearman's correlation coefficient was used. Logistic regression was used to calculate the predicted probability values for each marker separately and for all combinations of markers, and then the receiver operating characteristic (ROC) curves were constructed. The validity of suitable variables alone and in combination with screening tests for ovarian cancer was assessed using the area under the ROC curve. Serial and parallel analyses were conducted to explore the diagnostic value of variables for ovarian cancer. All statistical tests were two-sided, and differences were considered statistically significant at p-values < 0.05.

## 3. Results

### 3.1. Study Population Characteristics

Diagnostic samples from 173 patients with epithelial ovarian cancer and 70 healthy controls were used in our study. Detailed clinical information for patients is displayed in Supplemental Tables 1 and 2. From the 173 patient samples, there were 21 patients with very low CA125 concentration (lower than 35 U/ml). In our study, the low CA125 concentration group was defined as patients with a concentration equal to or below 35 U/ml, while the high CA125 concentration group possessed concentrations higher than 35 U/ml. There was no significant difference in pathological characteristics between these two groups (Supplemental Table 3). Comparison of the clinical parameters between the high CA125 concentration and low concentration groups is shown in Table 1.

According to the* t-*test results comparing high and low concentration group parameters, significant differences were found for the CA125 (*p*≦0.001), neutrophil (*p*=0.002), and NLR (*p*=0.002) parameters. Based on the Spearman analysis, low CA125 concentration in patients was also correlated with the number of neutrophils (r=0.24,* p*=0.001) and with NLR (r=0.239,* p*=0.002) (Table 2).

### 3.2. Diagnostic Significance of NLR and CA125 in Different Groups

**Figure 1:**
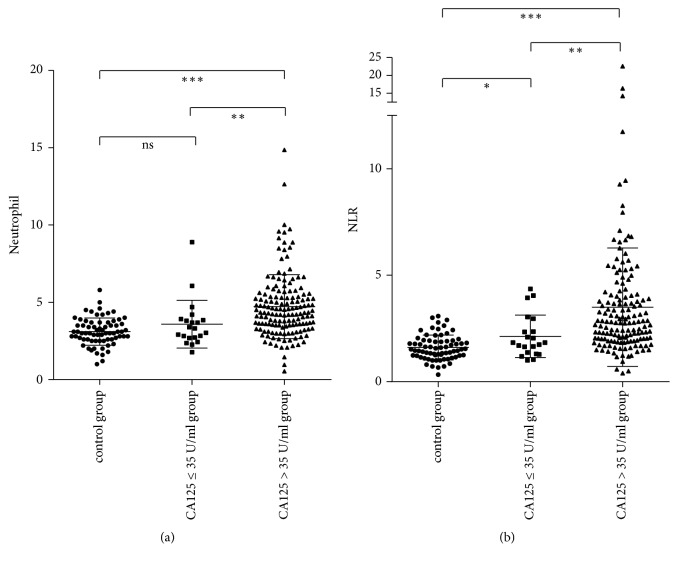
Neutrophil cell number (x 10^9^/L) and NLR in control (healthy people), low concentration (CA125≤ 35U/ml), and high concentration (CA125>35U/ml) groups. NLR in both low and high concentration groups was significantly higher than that in the control group. *∗* p<0.05, *∗∗* p<0.01, *∗∗∗* p<0.001.

To examine whether NLR is a valuable indicator for ovarian cancer patients, 70 healthy people were included as a control group. The baseline data for the control, low CA125, and high CA125 groups are shown in Supplemental Table 4. According to Figure 1, compared with the control group, there was a significant difference in NLR values found in both low and high concentration groups. However, for the neutrophil data, there was no difference between the control and low CA125 concentration groups. Based on these results, NLR could be another good indicator for patients with low CA125 concentrations.

**Figure 2:**
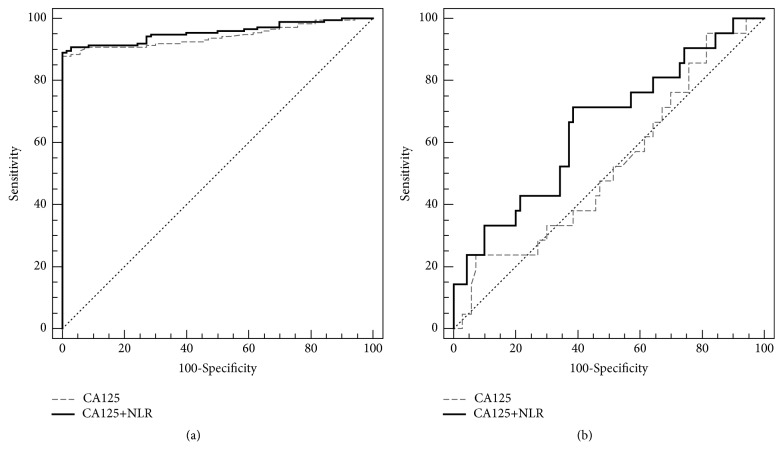
Receiver operating characteristic (ROC) curve for the entire patient group (a) or only the low CA125 concentration patient group (b).

To further compare the utility of NLR in ovarian cancer as an additional discriminative marker, ROC curves were used to analyze the entire patient group (including high and the low concentration patients) (Figure 2(a)) or only the low concentration group (Figure 2(b)). In the entire patient group, the area under the curve (AUC) for CA125 alone was 0.942, with a* p*-value < 0.0001, while the AUC for CA125 and NLR combined was 0.955, with a* p*-value < 0.0001. The AUC for CA125 was a little higher than that for CA125 alone, suggesting that the combination of CA125 and the NLR may be a good indicative maker for ovarian cancer prediction compared to CA125 alone, which is used widely now.

In the low concentration group, the AUC for CA125 alone was 0.524, but the* p-*value (0.7236) > 0.05, indicating CA125 alone is not a suitable marker. The AUC for the CA125 and NLR combined was 0.652, with a* p*-value (0.0323) < 0.05. Although the AUC for CA125 and NLR combined was not very large, it was still larger than that for CA125 alone and was statistically significant. Based on those results, we believe the CA125 and NLR combined may be a good predicator for the low CA125 concentration group (Table 3).

To categorize patients or healthy people as combined-marker positive or negative and to analyze these two different markers (CA125 alone and CA125 and NLR combined) to identify a good predicator for ovarian cancer screening, the optimal value at a cutoff value point was used. In the entire patient group, the combination marker CA125>24.55 U/ml and NLR >3.00 was used, and in the low concentration group, the combination marker CA125>7.65 U/ml and NLR >1.72 was used. The CA125 > 35 U/ml marker, which is widely used for tumor screening in routine physical examination tests, was used for both groups. A true positive or true negative was based on the pathology report of the patient. To evaluate these two different predictive makers, the sensitivity, specificity, positive predicative value (PPV), and negative predicative value (NPV) were calculated as shown in Table 4. All results are shown in Table 5.

In the entire patient group, CA125 > 35 U/ml yielded a sensitivity of 87.9% and specificity of 100%, with PPV equal to 100% and NPV equal to 76.9%. The combination marker was divided into two groups, CA125 > 24.55 U/ml and NLR>3.00 or CA125 >24.55 U/ml or NLR>3.00. Using the CA125 >24.55 U/ml and NLR>3.00 maker, a sensitivity of 38.2% and specificity of 100%, with PPV equal to 100% and NPV equal to 39.5%, were obtained. The CA125 >24.55 U/ml or NLR>3.00 group obtained a sensitivity of 92.5% and specificity of 78.6%, with PPV equal to 91.4% and NPV equal to 80.9%.

In the low concentration group, NPV was 76.9%, but the sensitivity, specificity, and PPV were 0%, indicating CA125> 35U/ml alone is strongly unsuitable as an ovarian cancer predicator. CA125 >7.65 U/ml and NLR>1.72 yielded a sensitivity of 52.4% and specificity of 67.1%, with PPV equal to 32.4% and NPV equal to 82.5%. The CA125 >7.65 U/ml or NLR>1.72 group obtained a sensitivity of 100% and specificity of 12.9%, with PPV equal to 25.6% and NPV equal to 100%.

## 4. Discussion

The high fatality rate for ovarian cancer patients is primarily due to a lack of early detection. Many patients are not diagnosed until late stage, so early detection for ovarian cancer is urgent. Routine physical examination is a good way to monitor our health, especially for early detection of cancer. Currently, the concentration of serum CA125 monitoring is the main method used for ovarian cancer screening during physical examination. In addition, in people who have been diagnosed with ovarian cancer, CA125 concentration can also be used to evaluate survival after treatment. Unfortunately, not all ovarian cancer patients exhibit high CA125 concentrations. In our study, 21 of 173 (approximately 12%) patients presented concentrations lower than 35 U/ml, despite being diagnosed with ovarian cancer by FIGO stage from I to IV (Supplemental Table 3). Our study only involved 173 patients, so if the study number was increased, we believe the percentage of low CA125 concentrations in cancer would be much larger. Hence, identifying a way to avoid these false negatives due to ovarian cancer cases with low CA125 concentration is very important. Because early cancer stage discovery is mainly based on routine physical examination, a cost-effective assay also should be considered.

Systemic inflammation is associated with many different cancers through induction of angiogenesis, metastasis, and cell proliferation [10]. NLR is an inflammatory marker used for evaluating patient status. For ovarian cancer, pretreatment NLR was elevated in epithelial ovarian cancer and exhibited predictive prognostic significance of survival after treatment. Immune complexes (ICs) are formed by the antigen and antibody against the antigen, and circulating immune complexes (CICs) are free ICs that circulate through body fluids. Normally, large size CICs can be cleared by macrophages, and small size CICs can be expelled from the body by the kidney filtration system. However, some middle size CICs cannot be cleared and remain in the circulatory system. These CICs could activate the inflammatory response, which is a basic mechanism for immune complex disease. In 2010, Daniel et al. demonstrated the existence of CICs involving CA125 and suggested that CA125 CICs provided an explanation for ovarian cancer with low CA125 concentrations [3]. Based on all of these findings, NLR should be a good indicator for ovarian cancer. In addition, because NLR results are very easily obtained, the cost-effectiveness is in accordance with the demands of screening markers for routine physical examination.

One hundred seventy-three ovarian cancer patients were involved in our study, and 21 of them exhibited low CA125 concentrations with no significant difference in age, lymphocyte number, or monocyte number. However, compared with the high concentration group, a significantly lower NLR was found in the low concentration group. According to the Spearman test results, high or low CA125 concentration for cancer patients was positively correlated with NLR. Thus, our study also demonstrates that NLR could be another good marker for ovarian cancer patients. Our ROC-AUC results proved that CA125 and NLR combined should be a good indicator for complete patient groups and for patients with low CA125 concentrations.

The marker we discussed herein is intended to be used for cancer screening during routine physical examination. In other words, positive markers should be maximally distinguished from the entire examination population, despite some false positives. Hence, the patients with the lowest levels would be missed by the marker. For further diagnosis of ovarian cancer and to exclude false positives, tests will be conducted by exams in the hospital, including CT and pathology. Hence, sensitivity and NPV should be the most important characteristics to be considered. The sensitivity rate for a marker is the true positive rate diagnosed by this marker, so the higher the sensitivity, the fewer the patients who will be missed. NPV is a negative predictive value equal to negatives diagnosed by the marker vs the true negatives, so the higher the NPV, the fewer the true positives left.

In the entire patient group, the ROC-AUC for CA125 and NLR (0.955) was a little larger than that for CA125>35 U/ml alone (Table 3), but the sensitivity was not as good as the traditional method, that is, CA125>35 U/ml alone. The sensitivity for the combination group was only 38.2%, while CA125>35 U/ml alone was up to 87.9%. However, for the CA125 or NLR group, both the sensitivity and the NPV were higher than those for the CA125>35 U/ml alone group, indicating that CA125 >24.55 U/ml or NLR>3.00 may be a better predictor for ovarian cancer screening during normal physical examination. NLR is an inflammatory maker correlated with many different malignant and nonmalignant diseases [11, 12]. Furthermore, the size of our sample was less than 200, and larger sample numbers and further study are needed to confirm whether CA125 >24.55 U/ml or NLR>3.00 can replace the traditional marker alone (CA125>35 U/ml).

For the low concentration group, we believe these results represent a new strategy for cancer screening during routine physical examination that may avoid missing ovarian cancer cases with low CA125 concentrations. Based on our data (Table 4), the CA125 > 35 U/ml group had zero sensitivity and zero specificity for ovarian cancer, indicating the traditional screening marker as useless in these cases. Although the CA125 or NLR group had 100% sensitivity and NPV, the specificity was too low at only 12.9%, which was not acceptable by clinical standards. In the CA125 and NLR groups, 52.4% sensitivity and 82.5% NPV were obtained with an acceptable sensitivity (67.1%). Based on these results, we believe that CA125>7.65 U/ml and NLR >1.72 may be a good additional discriminative marker for ovarian cancer screening in the population with CA125 lower than 35 U/ml. However, since the number of patients with ovarian cancer in this cohort was limited and all samples were from the oncology hospital, further confirmation will be needed using more samples from more diverse hospitals.

**Figure 3:**
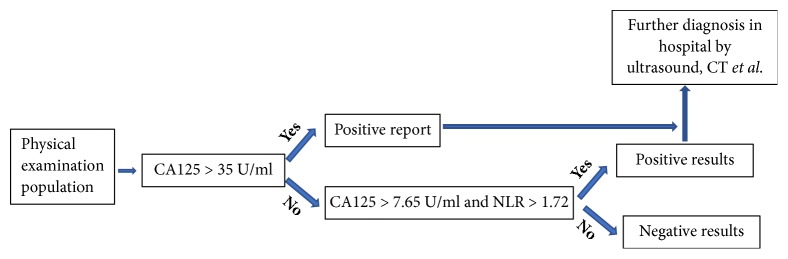
The illustration of two-step evaluation procedure for ovarian cancer screening.

In conclusion, based on our data, we believe that CA125>7.65 U/ml and NLR >1.72 would be an effective maker for patients with low CA125 concentration. To avoid missing a diagnosis of ovarian cancer due to a false negative during routine physical examination, a new strategy for cancer screening with two-step evaluation that is easy to conduct and cost-effective needs to be developed (Figure 3). However, using lower cutoff concentration for screening can lead to more diagnostic tests and, hence, higher cost of healthcare. Further studies will be needed to establish the outcome benefits of our suggested strategy.

## Figures and Tables

**Table 1:** Comparison of clinical parameters between high CA125 concentrations and low CA125 concentrations in epithelial ovarian cancer patients.

Parameters	CA125≤ 35 U/ml	CA125> 35 U/ml	*P* value
	(n = 21)	(n = 152)	
	Median (range)	Median (range)	
Age (years)	54.50 (23-71)	55.00 (24-80)	0.733
CA125 (U/mL)	15.88 (3.45-61.67)	534.40 (40.04-21269.00)	≦ 0.001
Neutrophil (x10^9^/L)	3.31 (1.78-8.89)	4.32 (0.54-14.86)	0.002
Lymphocyte (x10^9^/L)	1.87 (0.65-2.81)	1.61 (0.34-5.45)	0.074
Monocyte (x10^9^/L)	0.31 (0.21-0.63)	0.40 (0.02-1.36)	0.045
NLR	1.84 (1.01-4.35)	2.77 (0.40-22.57)	0.002

**Table 2:** Spearman correlation analysis.

	Neutrophil	NLR
r	0.24	0.239
*p*	0.001	0.002

**Table 3:** ROC-AUC for individual and combined variable analyses for ovarian cancers.

Variables	ROC-AUC	Cut-off	Value at cut-off point	*p*-value
*Healthy people vs the entire cancer patient group (including low and high CA125 concentrations)*				
CA125	0.942 (0.905-0.968)	0.5654	CA125 (33.84 *∗*)	< 0.0001
CA125 + NLR	0.955 (0.921-0.978)	0.6455	CA125 (24.55*∗*) NLR (3.00)	< 0.0001

*Healthy people vs the low CA125 concentration group*				
CA125	0.526 (0.418-0.632)	0.2546	CA125 (26.36*∗*)	0.7236
CA125 + NLR	0.652 (0.545-0.749)	0.1942	CA125 (7.68*∗*) NLR (1.72)	0.0323

Abbreviations. AUC: under the curve; ROC-AUC: areas under curves; *∗*: U/mL.

**Table 4:** The formula of the sensitivity, specificity, PPV, and the NPV.

		Pathology results (Gold standard)		Total
		Positive	Negative	
		(Cancer group)	(Healthy group)	
Analyzed marker	Positive	a (TP)	b (FP)	a+b
	Negative	c (FN)	d (TN)	c+d
Total		a+c	b+d	a+b+c+d

TP: true positive; FP: false positive; FN: false negative; TN: true negative.

Sensitivity=a/(a+c); specificity =d/(b+d); PPV=a/(a+b); NPV=d/(c+d).

**Table 5:** Characterization of the different diagnostic methods for different study groups.

	Positive#	Negative#	Total	Sensitivity	Specificity	PPV	NPV
*Entire patient group*							
CA125>35 U/ml							
Positive	152	0	152	87.9%	100%	100%	76.9%
Negative	21	70	91				
Total	173	70	243				
CA125 >24.55 U/ml and NLR>3.00							
Positive	66	0	66	38.2%	100%	100%	39.5%
Negative	107	70	177				
Total	173	70	243				
CA125 >24.55 U/ml or NLR>3.00							
Positive	160	15	175	92.5%	78.6%	91.4%	80.9%
Negative	13	55	68				
Total	173	70	243				

*Low concentration patient group*							
CA125>35 U/ml							
Positive	0	0	0	0%	0%	0%	76.9%
Negative	21	70	91				
Total	21	70	91				
CA125 >7.65 U/ml and NLR>1.72							
Positive	11	23	34	52.4%	67.1%	32.4%	82.5%
Negative	10	47	57				
Total	21	70	91				
CA125 >7.65 U/ml or NLR>1.72							
Positive	21	61	82	100%	12.9%	25.6%	100%
Negative	0	9	9				
Total	21	70	91				

PPV: positive predictive value; NPV: negative predictive value; #: results based on pathology test.

## Data Availability

The data used to support the findings of this study are included within the article and the supplementary information file(s).

## Supplementary Materials

Supplementary Materials Supplemental Table 1. Patient population characteristics. Supplemental Table 2. Overview on the WBC parameters, NLR, and some normal cancer screening tumor markers for 173 epithelial ovarian cancer patients. Supplemental Table 3. Comparison of pathological characteristics between high CA125 concentrations and low CA125 concentrations in epithelial ovarian cancer patients. Supplemental Table 4. Baseline data of the subjected patients.
