# Supplementary material for: Neutrophil-to-Lymphocyte Ratio in Ovarian Cancer Patients with Low CA125 Concentration
Source: Biomed Res Int. 2019 Jun 25;2019:8107906. doi: 10.1155/2019/8107906 (PMC6614957; doi:10.1155/2019/8107906)
Supplement: Supplementary Materials — Supplemental Table 1. Patient population characteristics. Supplemental Table 2. Overview on the WBC parameters, NLR, and some normal cancer screening tumor markers for 173 epithelial ovarian cancer patients. Supplemental Table 3. Comparison of pathological characteristics between high CA125 concentrations and low CA125 concentrations in epithelial ovarian cancer patients. Supplemental Table 4. Baseline data of the subjected patients. [file 8107906.f1.pdf]

Supplemental Table 1. Patient Population Characteristics.

Group	Average Age	n (%)
Healthy	45 (28-71)	70 (100)
Ovarian cancer	55 (23-80)	173 (100)
FIGO stage		
Stage Ⅰ		5 (2.9)
Stage Ⅱ		19 (11.0)
Stage Ⅲ		34 (19.7)
Stage Ⅳ		21 (12.1)
Grade		
Grade 1		11 (6.4)
Grade 2/3		67 (38.7)
Metastases		
Yes		63 (36.4)
No		110 (63.6)

Abbreviation: FIGO, The International Federation of Gynecology and Obstetrics.

Supplemental Table 2 Overview the WBC parameters、NLR and some normal cancer screening tumor markers for 173 epithelial ovarian cancer patients.

	Number of samples	Mean	Median	Range
Neutrophil (x10^9/L)	173	4.58	4.20	0.54-14.86
Lymphocyte (x10^9/L)	173	1.64	1.65	0.34-5.45
Monocyte (x10^9/L)	173	0.42	0.39	0.02-1.36
NLR	173	3.33	2.61	0.40-22.57
CA125 (U/mL)	173	944.90	410.80	3.45-21269.00
SCC (μg/L)	168	0.85	0.60	0.20-11.30
CEA (μg/L)	170	4.90	1.27	0.20-211.20
HCG (mIU/mL)	168	4.35	1.37	0.10-235.20
AFP (ng/mL)	167	9.36	2.51	0.61-1004.00
CA242 (U/mL)	99	93.08	6.23	0.58-3604.87
CA72-4 (U/mL)	84	64.83	6.58	0.41-2256.00
CA19-9 (U/mL)	82	202.50	9.75	0.60-13987.00

Supplemental Table 3 Comparison of pathological characteristics between high CA125 concentrations and low CA125 concentrations in epithelial ovarian cancer patients.

Characteristic	CA125≤ 35U/ml (n = 21)	CA125＞ 35U/ml (n = 152)	*P* value
Grade			
Grade 1	2 (9.5)	6 (3.9)	0.235
Grade 2/3	6 (28.6)	69 (45.4)	
Unknown	13 (61.9)	77 (50.7)	
FIGO stage			
Stage Ⅰ/Ⅱ	3 (14.3)	18 (11.8)	0.134
Stage Ⅲ/Ⅳ	3 (14.3)	55 (36.2)	
Unknown	15 (71.4)	79 (52.0)	
Metastases			
Yes	4 (19.0)	59 (38.8)	0.078
No	17 (81.0)	93 (61.2)	

Supplemental Table 4 Baseline data of the subjected patients.

Parameters	Healthy People Group (n = 70)	Low CA125 Group (n = 21)	High CA125 Group (n = 152)
Age (years)	45.00 (28-71)	54.50 (23-71)	55.00 (24-80)
CA125 (U/mL)	16.38 (1.56-33.84)	15.88 (3.45-61.67)	534.40 (40.04-21269.00)
Neutrophil ($x10^9$/L)	3.00 (1.00-5.80)	3.31 (1.78-8.89)	4.32 (0.54-14.86)
Lymphocyte ($x10^9$/L)	2.00 (0.70-4.20)	1.87 (0.65-2.81)	1.61 (0.34-5.45)
NLR	1.52 (0.33-3.08)	1.84 (1.01-4.35)*	2.77 (0.40-22.57)
